# 4541 Harnessing the Power of the Electronic Medical Record in Interstitial Lung Disease

**DOI:** 10.1017/cts.2020.178

**Published:** 2020-07-29

**Authors:** Erica Farrand, Eric Vittinghoff, Harold Collard

**Affiliations:** 1 University Of California, San Francisco

## Abstract

OBJECTIVES/GOALS: Harnessing the EHR to support clinical research is critical for the study of rare diseases such as interstitial lung disease (ILD). However no studies have compared EHR and research-quality data in the ILD population. Our objectives were to (1) identify ILD patients and extract clinical data from an EHR system and (2) assess the performance of ILD data capture. METHODS/STUDY POPULATION: Case validated algorithms were implemented to identify patients from the University of California San Francisco EHR and extract key ILD clinical information including, demographic variables, process measures and patient outcomes. Key clinical information were defined based on consensus statements and ILD clinical trials. A subset of ILD patients, had variables recorded in both the EHR and a separate ILD longitudinal research database. The completeness of EHR data capture and level of agreement were compared between three data collection methods: (1) data manually and systematically collected for an ILD research database (gold standard), (2) data automatically extracted from structured fields in the EHR, and (3) data extracted from unstructured data sources. RESULTS/ANTICIPATED RESULTS: We identified 5857 ILD patients in the EHR, of which 2100 patients had data available in the both the EHR and research database. Baseline demographic variables, co-morbidities, use of diagnostic testing, pharmacotherapy were accurately extracted from structured fields. Outcome measures, including lung physiology, radiographic patterns, pathology results, and health related quality of life (HRQoL) were unevenly extracted from structured fields alone. With the exception of HRQoL, these measures were accurately captured in unstructured EHR sources. Notably, certain metrics were better defined in the EHR, including health care resource utilization metrics, acute exacerbations, medication side effects, supplemental oxygen use and specialty care referrals (rheumatology, lung transplant, palliative care, etc). DISCUSSION/SIGNIFICANCE OF IMPACT: A large real-world ILD cohort can be algorithmically extracted from the EHR along with key clinical variables with accuracy comparable to protocol-driven research databases. Rigorous assessment of the types of disease-specific variables that are present in EHR-derived data will inform future interventions to improve the fidelity, accessibility and use of the EHR in clinical research.

